# Computational modeling of the gut microbiota reveals putative metabolic mechanisms of recurrent *Clostridioides difficile* infection

**DOI:** 10.1371/journal.pcbi.1008782

**Published:** 2021-02-22

**Authors:** Michael A. Henson

**Affiliations:** Department of Chemical Engineering and Institute for Applied Life Sciences, University of Massachusetts, Amherst, Massachusetts, United States of America; Christian Albrechts Universitat zu Kiel, GERMANY

## Abstract

Approximately 30% of patients who have *Clostridioides difficile* infection (CDI) will suffer at least one incident of reinfection. While the underlying causes of CDI recurrence are poorly understood, interactions between *C*. *difficile* and commensal gut bacteria are thought to play an important role. In this study, an *in silico* pipeline was used to process 16S rRNA gene amplicon sequence data of 225 stool samples from 93 CDI patients into sample-specific models of bacterial community metabolism. Clustered metabolite production rates generated from post-diagnosis samples generated a high *Enterobacteriaceae* abundance cluster containing disproportionately large numbers of recurrent samples and patients. This cluster was predicted to have significantly reduced capabilities for secondary bile acid synthesis but elevated capabilities for aromatic amino acid catabolism. When applied to 16S sequence data of 40 samples from fecal microbiota transplantation (FMT) patients suffering from recurrent CDI and their stool donors, the community modeling method generated a high *Enterobacteriaceae* abundance cluster with a disproportionate large number of pre-FMT samples. This cluster also was predicted to exhibit reduced secondary bile acid synthesis and elevated aromatic amino acid catabolism. Collectively, these *in silico* predictions suggest that *Enterobacteriaceae* may create a gut environment favorable for *C*. *difficile* spore germination and/or toxin synthesis.

## Author summary

*Clostridioides difficile* is an opportunistic human pathogen responsible for acute and sometimes chronic infections of the colon. Elderly individuals who are immunocompromised, frequently hospitalized and antibiotic recipients are particular susceptible to *C*. *difficile* infection (CDI). Approximately 30% of CDI patients will suffer at least one episode of reinfection, commonly termed recurrence. The objective of the current study was to utilize computational metabolic modeling to investigate the hypothesis that recurrent infections are related to the composition of the gut bacterial community within each patient. Our modeling results suggest that patients who have high abundances of the bacterial family *Enterobacteriaceae* following antibiotic treatment are more likely to develop recurrent infections due to a metabolically-disrupted gut environment. Successful treatment of recurrent patients with transplanted fecal matter is computationally predicted to correct this metabolic disruption, suggesting that interactions between *C*. *difficile* and *Enterobacteriaceae* are worthy of additional study.

## Introduction

The anaerobic bacterium *Clostridioides difficile* is an opportunistic pathogen responsible for infections of the human colon [1]. *C*. *difficile* infection (CDI) is most common in elderly patients previously treated with broad spectrum antibiotics that disrupt the healthy gut microbiota and produce a dysbiotic environment conducive to *C*. *difficile* germination, expansion and pathogenicity [2,3]. CDI has become particularly common in hospital settings due to the ability of *C*. *difficile* to form spores that adhere to surfaces and resist common disinfectant protocols. Some *C*. *difficile* strains have developed resistance to common antibiotics while also exhibiting more severe pathogenicity [4]. Studies estimate that 500,000 CDI cases occur in the U.S. annually [5], resulting in 29,000 deaths and over $4.8 billion in associated costs in acute care facilities alone [6].

Approximately 10% of healthy adults are asymptomatically colonized with *C*. *difficile* [7–9]. Commensal bacterial species in the healthy gut can provide resistance against *C*. *difficile* pathogenic colonization through a variety of metabolic mechanisms, including competition for dietary nutrients such as carbohydrates and amino acids [10] and conversion of host-derived primary bile acids that promote *C*. *difficile* spore germination to secondary bile acids that inhibit germination and growth [11]. Recurrence is a major challenge associated with CDI treatment, as approximately 30% of patients develop at least one occurrence of reinfection [12]. The host-microbiota mechanisms underlying recurrence are not well understood, as microbiota composition alone is a poor predictor of patient recovery versus recurrence [13–15]. For patients who suffer from repeated episodes of recurrence, fecal microbiota transplantation (FMT) is the last resort treatment. Despite its remarkable success rate approaching 90% [16], FMT remains controversial [17] as the donor microbiota confer poorly understood functions to the endogenous community [18] and may contain pathogenic strains not recognized during screening of donor stool [19].

The advent of high-throughput technologies such as 16S rRNA-encoding gene sequencing has yielded unprecedent insights into the composition of *in vivo* bacterial communities [20–22]. Despite numerous 16S-based studies that have attempted to correlate CDI disease state to gut bacterial composition [13,23–26], we still do not understand why some exposed individuals develop CDI while other individuals are asymptomatic [9,27,28] and why some infections become recurrent while other infections are effectively treated with antibiotics [29–32]. Furthermore, microbial communities being transplanted with FMT are poorly understood both with regard to their composition and the health-promoting metabolic functions being introduced [33–35]. Uncertainty at this level can decrease therapeutic efficacy and increase the risk of adverse events [19,36].

Translating composition data derived from 16S gene sequencing into an understanding of community function is a challenging problem. Gut bacteria often possess overlapping metabolic functions, such as their ability to synthesize secondary bile acids [37–39] and short-chain fatty acids like butyrate and propionate [40,41]. Furthermore, numerous studies [42–47] have demonstrated that microbiota composition is an individual characteristic and usually an inadequate measure for assessing healthy versus disease states. These critical gaps in knowledge exist because bacterial composition data alone is insufficient to characterize the metabolic state of the diseased gut and nutritional environments that are protective against CDI. The next step in microbiome research needs to be the translation of composition data into quantitative information about bacterial community dynamics and function [48–50].

In this study, a recently developed *in silico* modeling pipeline [51] was applied to the problem of identifying putative microbiota-based determinants of recurrent CDI. The pipeline was used to translate 16S-derived taxa abundances from stool samples of CDI and FMT patients into sample-specific models to quantify the metabolic capabilities of the modeled communities, which have been shown to correlate with clinical states in other microbiota-based disease processes [52,53]. The metabolic modeling approach can be viewed as complementary to more established analysis techniques such as Phylogenetic Investigation of Communities by Reconstruction of Unobserved States (PICRUSt; [54]) where 16S sequence data are mapped to a set of reference genomes/metagenomes of a large number of sequenced organisms to estimate the genomic content of the individual samples. While PICRUSt provides functional gene counts for each sample, the method does not allow for the prediction of community interactions such as nutrient competition and metabolite crossfeeding necessary for quantitative analysis of community metabolism. This study demonstrates the power of genome-scale metabolic modeling for distinguishing CDI disease states according to the metabolite synthesis capabilities of the individual communities.

## Materials and methods

### Patient data

Gut microbiota composition data were obtained from two published studies [13,55] in which patient stool samples were subjected to 16S rRNA gene amplicon library sequencing. The first study [13] included 225 longitudinal samples from 93 CDI patients ranging in age from 18 to 89 years. Each patient was characterized as either: *nonrecurrent* if a non-reinfected sample was collected >14 days after a previous *C*. *difficile* positive sample; *recurrent* if a positive sample was collected 15–56 days after a previous positive sample; and *reinfected* if a positive sample was collected >56 days after a previous positive sample (Table 1). Because patients in both groups were ultimately reinfected, the recurrent and reinfected patients were lumped together in this study and termed recurrent. The sample was defined as an *index* sample if it returned the first *C*. *difficile* positive for that patient, a *pre-index* sample if it was collected before the index sample, and *post-index* sample if it was collected after the index sample. Some patients (10/51 recurrent and 15/42 nonrecurrent) had received antibiotics before collection of the index sample, while all patients received a standard 14 day antibiotic treatment following a positive sample. Therefore, post-index samples collected within 14 days of a positive test overlapped with antibiotic treatment (S1 Table). The second study [55] used for community modeling included 40 samples from 14 FMT patients and 10 of their stool donors (Table 1).

**Table 1:** Summary of CDI patient data [13] and FMT patient data [55].

CDI patient data				FMT patient data	
	Nonrecurrent	Recurrent	Total		Total
Patients	42	51	93	Patients	14
Pre-index samples	1	5	6	Pre-FMT samples	14
Index sample	42	51	93	Donor samples	10
Post-index samples	37	89	126	Post-FMT samples	26
Total samples	80	145	225	Total samples	40

The 16S rRNA OTU reads available in the two original studies were generally at the genus and family taxonomic levels. These reads were mapped into taxa abundances for development of sample-specific community metabolic models. Using the 100 most abundant OTUs across the samples in each study, taxa abundances were derived as follows: (1) all OTUs belonging to the same taxonomic group were combined; (2) OTUs belonging to higher taxonomic groups (i.e. order and above) were eliminated to maintain modeling at the genus and family levels; and (3) the reduced set of OTUs was normalized such that the abundances of each sample summed to unity. To quantify the elimination of higher-level taxa, the total reads from step 3 were divided by the total reads of the 100 most abundant OTUs to generate an unnormalized total abundance for each sample. For the CDI dataset, this procedure resulted in 48 taxa (40 genera, 8 families) that accounted for an average of 97.7% of the top 100 OTU reads across the 225 samples (S1 Table). Due to the non-negligible abundance of the class Gammaproteobacteria in the FMT dataset (average abundance of 3.4%), this class was retained to generate 39 taxa (30 genera, 8 families, 1 class) that accounted for an average of 99.3% of the top 100 OTU reads across the 40 samples (S2 Table).

### Community metabolic modeling

The computational modeling workflow involved translating the normalized taxa abundance data into sample-specific community metabolic models for analysis of metabolite production capabilities (S1 Fig). Taxa represented in the normalized CDI and FMT samples were modeled using genome-scale metabolic reconstructions from the Virtual Metabolic Human (VMH) database [56]. The function createPanModels within the metagenomics pipeline (mgPipe, [51]) of the MATLAB Constraint-Based Reconstruction and Analysis (COBRA) Toolbox [57] was used to create higher taxa models from the 818 strain models available in the VMH database. The sample taxa were mapped to these pan-genome models according to their taxonomy (e.g. Clostridium cluster XI containing *C*. *difficile* was mapped to the family *Peptostreptococcaceae*). The function initMgPipe was used to construct a community metabolic model for each of the 225 CDI and 40 FMT samples. Model construction required specification of taxa abundances for each sample and maximum uptake rates of dietary nutrients, which was specified according to an average European diet (S3 Table, [53]).

The community models contained an average of 33,773 reactions (minimum 8,302; maximum 59,923) for the CDI samples and 36,278 reactions (minimum 26,466; maximum 46,179) for the FMT samples. All models contained the same constraints for the maximum nutrient uptake rates, while each model had different constraints imposed for the sample taxa abundances. mgPipe performed flux variability analysis (FVA) for each model with respect to each of the 411 metabolites assumed to be exchanged between the microbiota and the lumen and fecal compartments. FVA calculations were performed to either maximize the production of the metabolite or to minimize the uptake of the metabolite subject to the additional constraint that the community biomass flux remained in the range 0.4–1.0 mmol/day [53]. The FVA results were used within mgPipe to compute the net maximal production capability (NMPC, [51]) of each metabolite by each model (S4 Table for CDI; S5 Table for FMT) as a measure of community metabolic capability. Each NMPC value was calculated as the difference between two FVA solutions, the first which maximized metabolite secretion into the fecal compartment and the second which minimized metabolite uptake from the lumen compartment.

While a common problem in genome-scale metabolic modeling is the existence of ATP-producing futile cycles, this issue has received relatively little attention in the context of community metabolic models. To evaluate possible ATP-producing futile cycles and their impact on calculated NMPCs within mgPipe, a community model consisting of 200 randomly chosen VMH strains was constructed and constrained to have no nutrient uptakes (i.e. a closed model). When the ATP demand flux of each community strain was individually maximized, most strains generated a very small ATP flux (maximum = 0.01 mmol/gDW/h, minimum = 0 mmol/gDW/h, average = 0.0046 mmol/gDW/h). When these ATP-producing futile cycles were eliminated by changing the input parameter *u* in the mgPipe function createPersonalizedModel from *u* = 0.01 to *u* = 0, none of the 200 community strains were capable of generating a non-zero ATP demand flux. Critically, the value of the input parameter u was found to have no effect on the metabolite exchange calculations as all NMPCs were zero for both *u* values. Therefore, we concluded that the calculated NMPCs presented here could not be affected by the existence of possible ATP-producing futile cycles.

### Data analysis

Patient data consisted of normalized taxa abundances and model data consisted of calculated net maximal production capabilities (NMPCs), both of which could be connected to associated metadata on a sample-by-sample basis (S1 and S2 Tables). Both types of data were subjected to unsupervised machine learning techniques including clustering and principal component analysis (PCA) to extract putative relationships between partitioned samples/patients and clinical parameters such as recurrence. Rather than apply supervised learning to samples partitioned on their known clinical status (i.e. recurrent, nonrecurrent), unsupervised learning was performed to determine if samples clustered by taxa abundances or model-generated NMPCs could predict recurrence. This approach was applied under the hypothesis that clustering could partially unravel the complex CDI disease etiology and reveal at least one cluster with statistically high levels of recurrence or nonrecurrence. Clustering was performed using the MATLAB function kmeans with the squared Euclidean distance metric [58], the k-means++ algorithm for cluster center initialization [59] and 1,000 replicates. When clustering was applied to normalized taxa abundances of the CDI index samples, the Davies-Bouldin criterion [60] indicated that three clusters was the optimal number. To facilitate subsequent comparisons, three clusters also were used for other CDI datasets including the abundance post-index samples, the model-predicted index samples and the model-predicted post-index samples. When applied to taxa abundances of the FMT samples, the Davies-Bouldin criterion consistently indicated that the optimal cluster number was equal to the maximum number of clusters allowed. Instead, two clusters were used for the 40 FMT samples such that each cluster would contain a sufficiently large sample number to perform statistical analyses. For each case tested, the clustering method proved robust in that the same clustered samples were consistently returned despite the randomness of cluster initialization and the existence of local minima [61].

PCA was performed directly on normalized taxa abundance and calculated NMPC data rather than on data preprocessed with sample dissimilarity measures such as the Bray–Curtis [62] or UniFrac [63] metrics. This approach was deemed sufficient since PCA was used for preliminary data visualization and not quantitative data analysis. Statistical significance of associations between categorial variables (e.g. recurrent/nonrecurrent) across samples/patient groups were assessed using Fisher’s exact test [64]. Correlations between taxa based on their abundances across samples/patients were calculated using the proportionality coefficient [65], which accounts for the effects of data normalization. Statistically significant differences between metabolite NMPCs across samples/patients were assessed using the Wilcoxon rank-sum test [66]. The resulting p-values were used to calculate the false-positive discovery rate (FDR) for each metabolite using the MATLAB function mafdr with the Benjamini-Hochberg method [67].

## Results

### Clustered index samples were not associated with recurrence

**Fig 1:**
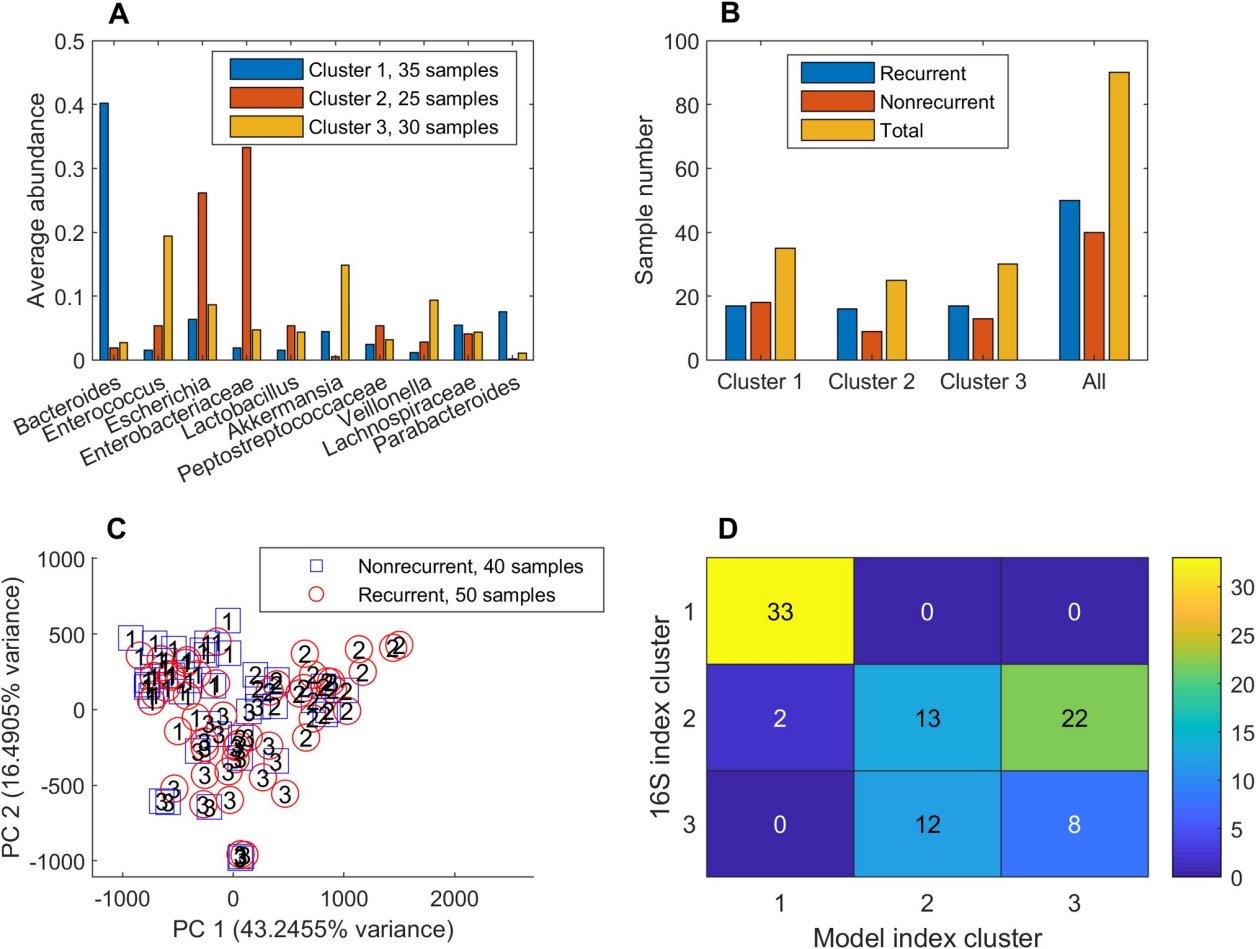
Clustering of 90 index samples using model-predicted metabolic capabilities. (A) Average taxa abundances across the samples in each cluster for taxa which averaged at least 5% of the total abundance. (B) Number of recurrent, nonrecurrent and total samples in each cluster and all 90 index samples. None of the clusters contained a disproportionate number of recurrent samples (Fisher’s exact test, p > 0.25). (C) PCA plot of the abundance data with each recurrent and nonrecurrent sample labeled by its associated cluster number. (D) Intersection between samples clustered based on model-predicted metabolic capabilities and 16S-derived abundance data. The number in each box represents the number of shared samples between clusters.

The CDI dataset [13] included 93 index samples (Table 1), which were defined as samples that returned the first *C*. *difficile* positive for each patient. The 90 index samples remaining after removal of three samples (2 nonrecurrent, 1 recurrent) containing less than 90% of modeled taxa were clustered using their model-predicted metabolic capabilities (i.e. NMPCs). Clustering was performed within MATLAB using the kmeans method with three clusters (see Materials and Methods) and generated a silhouette value of 0.42. The index samples were clustered into 25 samples with elevated *Enterobactericeae* and *Escherichia*, 30 samples with elevated *Enterococcus* and *Akkermansia*, and 35 samples dominated by *Bacteroides* (Fig 1A). None of the clusters exhibited a higher proportion of recurrent samples (p > 0.25; Fig 1B), and PCA showed no distinct structure with respect to recurrent/nonrecurrent samples (Fig 1C).

Similar analyses were applied to the normalized taxa abundances of the 90 index samples to determine if community composition would be more strongly associated with recurrence than predicted metabolic capabilities. The index samples were partitioned with three clusters (silhouette value = 0.49) into 20 samples with elevated *Enterobactericeae* and *Enterococcus*, 33 samples dominated by *Bacteroides*, and 37 samples with elevated *Escherichia* and *Akkermansia* (S2A Fig). While the *Enterobactericeae*/*Enterococcus* cluster exhibited a higher proportion of recurrent samples than the other two clusters and the entire index dataset (S2B Fig), none of these differences were significant (Fisher’s exact test, p > 0.5). When the index samples were analyzed with PCA, the abundance data exhibited structure with respect to the three clusters but not with respect to recurrent/nonrecurrent samples (S2C Fig). The taxa abundances and predicted metabolic capabilities generated different clusters, demonstrating that the community model inputs and outputs provided distinct information (Fig 1D). Therefore, the index samples, which were collected prior to standard CDI antibiotic treatment, were deemed to have no statistical association with recurrence.

### Post-index samples clustered by metabolic capability were associated with recurrence

The 119 post-index samples remaining after removal of seven samples (1 nonrecurrent, 6 recurrent) containing less than 90% of modeled taxa were clustered by their taxa abundances. The three clusters (silhouette value = 0.45) contained 15 samples dominated by *Enterobactericeae* with low abundances of *Bacteroides*, *Enterococcus* and *Escherichia*, 18 samples dominated by *Enterococcus* with low abundances of *Bacteroides*, *Escherichia* and *Enterobactericeae*, and 86 samples more diversely distributed (inverse Simpson index of 12.4 versus 2.2 and 1.9, respectively, for the other two clusters) and not dominated by a single taxa (S3A Fig). The high *Enterobactericeae* abundance cluster contained a disproportionate large number of recurrent samples (14/15) compared to the high *Enterococcus* cluster (9/18; Fisher’s exact test, p = 0.009; S3B Fig). In terms of classification capability, the high *Enterobactericeae* abundance cluster offered high precision with positive predictive value (PPV) = 0.93 but poor sensitivity with true positive rate (TPR) = 0.17. In other words, a sample contained in this cluster was very likely to be recurrent (14/15) but many recurrent samples (69/83) were not contained in this cluster. The recurrent samples in the high *Enterobactericeae* abundance cluster were clearly distinguishable in a PCA plot of the post-index abundance data (S3C Fig).

**Fig 2:**
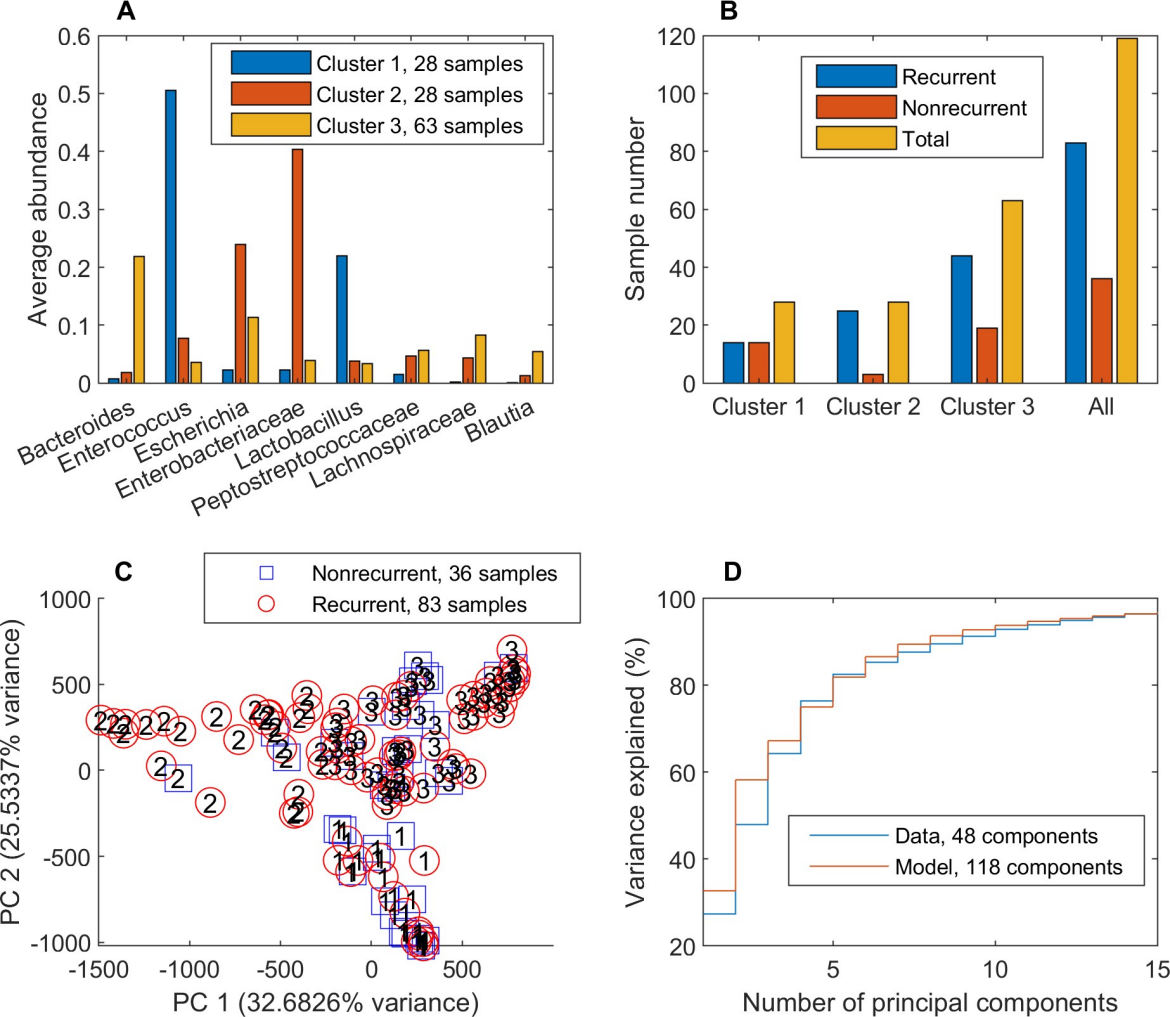
Clustering of 119 post-index samples using model-predicted metabolic capabilities. (A) Average taxa abundances across the samples in each cluster for taxa which averaged at least 5% of the total abundance. (B) Number of recurrent, nonrecurrent and total samples in each cluster and all 119 post-index samples. Cluster 2 contained a disproportionate number of recurrent samples (25/28) compared to the cluster 1 (14/28; p = 0.003) and the entire post-index dataset (83/119; p = 0.035). (C) PCA plot of model-predicted metabolic capabilities with each recurrent and nonrecurrent sample labeled by its associated cluster number. (D) Variance explained by PCA of 16S-derived abundance data and model-predicted metabolic capabilities. The total number of components for each dataset shown in the legend was determined by the MATLAB function pca.

NMPCs calculated for the 119 post-index samples were clustered to explore the hypothesis that metabolic outputs of the community models would be more strongly associated with recurrence than was possible with community compositions alone. These model-predicted metabolic capabilities were partitioned with three clusters (silhouette value = 0.34) into 28 samples with elevated *Enterobactericeae* and *Escherichia*, 28 samples with elevated *Enterococcus* and *Lactobacillus*, and 63 samples with elevated *Bacteroides* and more diversely distributed (inverse Simpson index of 11.6 versus 4.3 and 3.2, respectively, for the other two clusters; Fig 2A). The high *Enterobactericeae* cluster contained a disproportionate number of recurrent samples (25/28) compared to the high *Enterococcus* abundance cluster (14/28; p = 0.003) and the entire post-index dataset (83/119; p = 0.035; Fig 2B). Compared to the classification capability of the high *Enterobactericeae* cluster derived directly from abundance data, the model-derived cluster had slightly lower precision PPV = 0.89 but higher sensitivity TPR = 0.30.

The number of recurrent samples contained in the model-derived, high *Enterobactericeae* abundance cluster relative to the total number of recurrent post-index samples (25/83) compared to those in the abundance-based, high *Enterobactericeae* cluster (14/83) was not quite statistically disproportionate (p = 0.066). However, metabolic modeling did generate a larger cluster compared to direct use of abundance data (25 samples from 19 recurrent patients versus 14 samples from 10 recurrent patients), allowing more recurrent samples/patients for subsequent statistical analyses. The high recurrence *Enterobactericeae* cluster was distinguishable in the upper left quadrant of a PCA plot of the model-predicted metabolic capabilities due to the unique properties of these clustered samples (Fig 2C), an issue explored below in detail. Despite having 118 identified PCA components (less than the 411 possible components due to matrix conditioning) compared to the abundance data with 48 possible components, the model output data was more efficiently compressed with a small number of principal components (e.g. 58.2% versus 48.0% variance captured for two components; Fig 2D). This result suggested that the model-calculated NMPCs represented a more efficient set of data features than the 16S-derived abundances. Comparison of the three clusters generated with taxa abundances and with those generated with NMPCs generated a Rand value of 0.72, showing that two sets of CDI sample clusters were similar but also had numerous samples clustered differently. Collectively, these analyses demonstrated the potential benefits of model-predicted metabolic capabilities to quantify functions of bacterial communities rather than relying on community compositions alone.

The number of clusters was varied to further explore partitioning of the 119 model-predicted post-index samples. Interestingly, two clusters also produced a relatively small group with elevated *Enterobactericeae* and *Escherichia* (34 samples) as well as generating a second, larger group with elevated *Enterococcus*, *Bacteroides* and *Lactobacillus* (85 samples; S4A Fig). As the number of clusters was increased, the *Enterobactericeae*/*Escherichia* group split into two separate clusters and the *Enterococcus*/*Bacteroides*/*Lactobacillus* group split into three separate clusters (S4B–S4E Fig). The high *Enterobactericeae* abundance clusters retained their property of disproportionately high recurrence compared to the *Enterococcus*-elevated clusters for all cases (p < 0.04; S4F Fig), suggesting a possible role for *Enterobactericeae* in CDI recurrence during antibiotic treatment.

### Clustered post-index samples were predicted to exhibit distinct bile acid and aromatic amino acid metabolism

NMPCs calculated for the 119 post-index samples with respect to each of the 411 exchanged metabolites were statistically analyzed to assess metabolic differences between model-derived clusters. The Wilcoxon rank sum test was applied to the NMPCs across all samples in two chosen clusters on a metabolite-by-metabolite basis. To reduce the number of reported metabolites, statistically different metabolite NMPCs (FDR < 0.05) also were required to have an average NMPC > 50 mmol/day in at least one cluster and an average NMPC that differed between the clusters by at least 100%. The high *Enterobactericeae* abundance cluster (HEb, 28 samples) and the high *Bacteroides* abundance cluster (HBo, 63 samples) had 47 differentially produced metabolites (S5 Fig, S6 Table), including 7 metabolites associated with AAA degradation elevated in the HEb cluster. A comparison of the HEb cluster and the high *Enterococcus* abundance cluster (HEc, 28 samples) generated 44 differentially produced metabolites (S6 Fig, S6 Table), including 19 metabolites associated with aromatic amino acid (AAA), bile acid (BA) and butanoate metabolism. Interestingly, 11 secondary BA metabolites were elevated in the HEc cluster compared to the HBo cluster, accounting for 25% of the differentially produced metabolites (S7 Fig, S6 Table).

**Fig 3:**
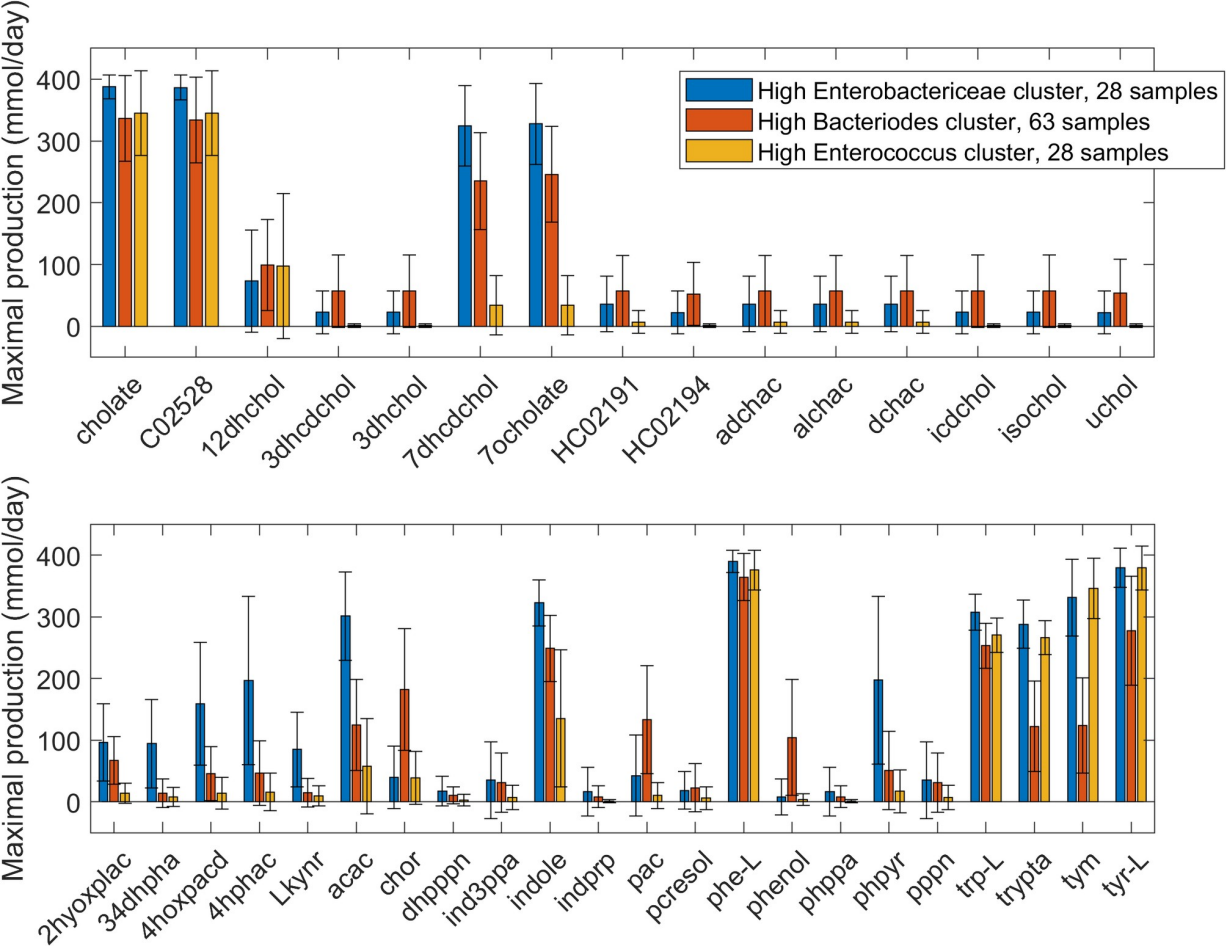
Net maximal production rates of bile acid and aromatic amino acid metabolites in the high *Enterobacteriaceae*, high *Bacteroides* and high *Enterococcus* abundance clusters generated from 119 model-predicted post-index samples. (A) Bile acid metabolites in which the average production rate was non-zero in at least one cluster. (B) Aromatic amino acid metabolites in which the average production rate was non-zero in at least one cluster. Error bars represent standard error of the mean. Metabolites abbreviations are taken from the VMH database (www.vmh.life). Full metabolite names, their associated metabolic pathways and numeric values for their average production rates in each cluster are given in S6 Table.

Due to their differential utilization across the three clusters, the BA and AAA pathways were examined more carefully by collecting metabolites belonging to these pathways that were allowed to be exchanged according to the metabolic models. The HEb cluster was predicted to have the highest production capabilities for the two unconjugated primary BAs (Fig 3A), which have been reported to either promote (cholate) or inhibit (chenodeoxyholate, C02528) *C*. *difficile* germination [68,69]. By contrast, the HBo cluster generated the highest production of most secondary BAs, which are known to be generally protective against CDI [2,70,71]. Interestingly, the HEc cluster had much lower production capabilities for secondary BAs. The HEb cluster was predicted to generate higher production of metabolites involved in AAA catabolism but not significantly higher production of the AAAs themselves (Fig 3B, S6 Table). Although positively correlated with the normalized *Enterobacteriaceae* abundance, the total synthesis rate of AAA catabolites was predicted to be a complex metabolic function of the community composition as samples with relatively low *Enterobacteriaceae* abundance could generate substantially higher AAA catabolite synthesis rates than samples with relatively high *Enterobacteriaceae* abundance. This AAA degradation ability was decreased in the HBo cluster and substantially lower in the HEc cluster, with the notable exceptions of the tyrosine degradation product tyramine (tyr) and the tryptophan-derived metabolite tryptamine (trypta). Interestingly, the AAA precursor chorismate (chor) was significantly elevated in the HBo cluster, yet the production capabilities of the AAA themselves were reduced in this cluster. Since the HEb cluster contained a disproportionately large number of recurrent samples compared to the other two clusters, these predictions suggest a possible role for AAA metabolism in recurrent CDI.

### Transient presence in the high Enterobactericeae abundance cluster was sufficient for elevated patient recurrence

**Fig 4:**
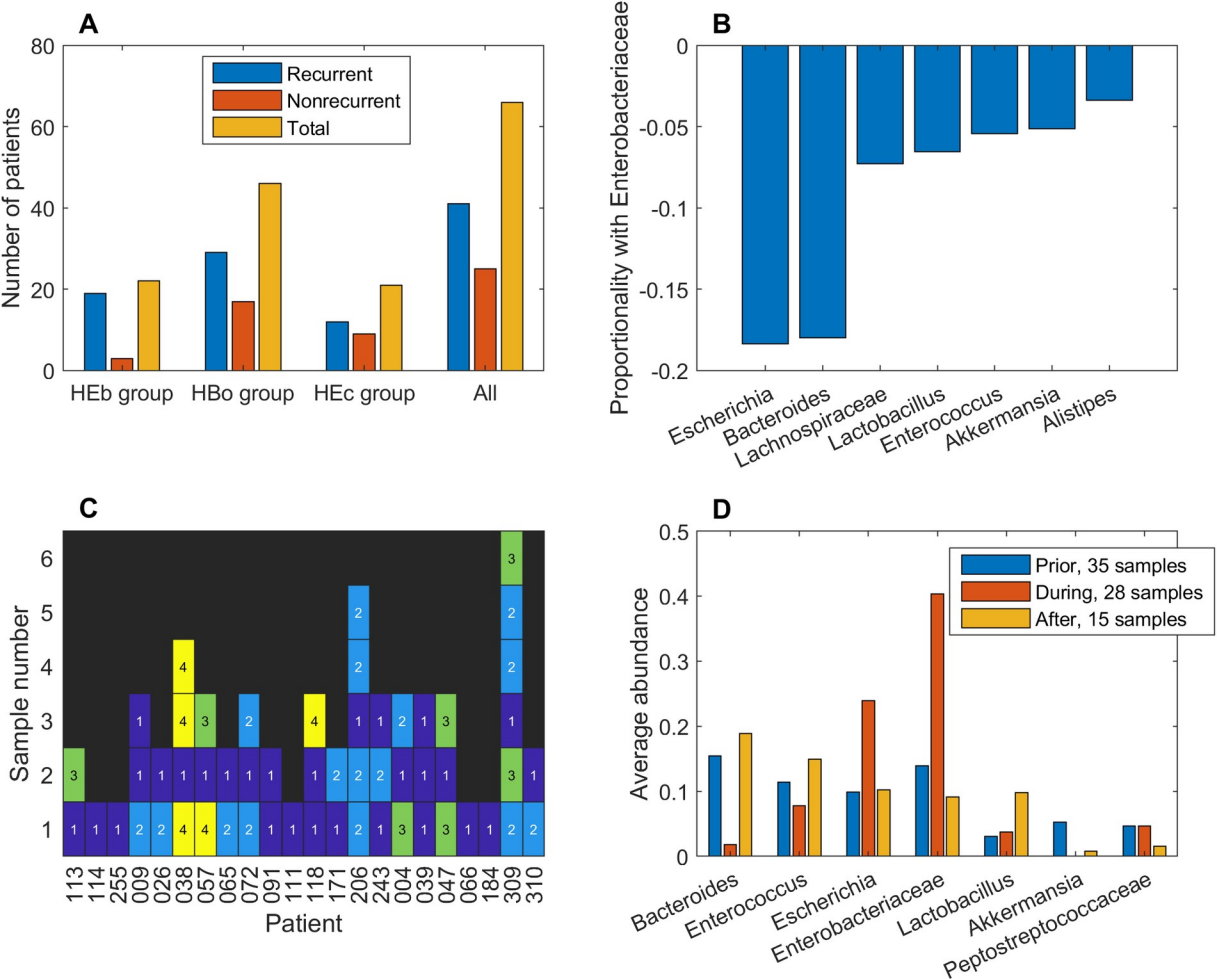
Analysis of patient samples in the high *Enterobacteriaceae* abundance (HEb) group. All post-index samples from the 22 patients with at least one sample in the high *Enterobacteriaceae* (HEb) abundance cluster were grouped to generate the enlarged HEb group of 55 samples. (A) The number of recurrent, nonrecurrent and total patients in the HEb group compared to those in the HBo and HEc groups. All post-index samples of the 46 patients represented in the HBo cluster and the 21 patients represented in the HEc cluster were grouped to produce the 87 and 47 samples, respectively, in the HBo and HEc groups. The 66 total patients represented by these samples were allowed to reside in multiple groups. (B) Correlation between *Enterobacteriaceae* and other taxa in the HEb group calculated from the 55 post-index samples as measured by the proportionality coefficient ρ. The 7 taxa with the largest |ρ| values are shown. (C) Transient progression of samples from the 22 patients in the HEB group with samples denoted as 1 if contained in the HEb cluster, 2 if contained in the HBo cluster, 3 if contained in the HEc cluster and 4 if not clustered due to low abundance of modeled taxa (see Methods). (D) Average taxa abundances for an expanded HEb group that also contained pre-index and index samples to generate a dataset of 78 samples. These samples were partitioned into 35 samples prior to patients entering the HEb cluster, 28 samples during patient presence in the HEb cluster and 15 samples after patients left the HEb cluster.

The HEb cluster contained a disproportionately large number of recurrent samples (25/28). To investigate the transient bacterial communities of the 22 patients from which these samples were collected, all post-index samples from these patients were grouped to generate an enlarged dataset of 55 samples. Similarly, all post-index samples from the 46 patients in the HBo cluster and the 21 patients in the HEc cluster were grouped to generates datasets containing 87 and 47 samples, respectively. The 66 total patients represented by these samples were allowed to reside in multiple datasets referred to as the HEb, HBo and HEc groups. The HEb group contained a disproportionately large number of recurrent patients (19/22) compared to the HEc group (12/21; p = 0.045) and all grouped patients (41/66; p = 0.038; Fig 4A). Within the HEb group, *Enterobacteriaceae* was most negatively correlated with *Escherichia* and *Bacteroides* (proportionality coefficient ρ = -0.18 for both pairs; Fig 4B). In addition to health-promoting *Bacteroides* [72], *Enterobacteriaceae* was negatively correlated with other taxa including *Lachnospiraceae* [73], *Lactobacillus* [74] *Akkermansia* [75] and *Alistipes* [74] reported to be protective against CDI.

Taxa abundances from the HEb group showed considerable dissimilarity between samples from an individual patient (Yue and Clayton dissimilarity index θ = 0.35 across the 22 patients; S8 Fig), with the only consistent feature among the 22 patients being at least one sample contained in the HEb cluster (Fig 4C). The first 3 patients (113, 114, 255) were nonrecurrent despite having a sample in the HEb cluster. Of the remaining 19 recurrent patients, 10 patients also had a sample in the HBo cluster and 4 patients also had a sample in the HEc cluster. Moreover, 7 of the 19 recurrent patients had final samples contained in either the HBo or HEc cluster. Given the irregular sampling frequency reported in the original clinical study [13], this analysis suggested that transient presence in the HEb cluster was sufficient for a patient to have an elevated risk of recurrence.

To investigate if the bacterial community within an individual patient showed distinct trends once the HEb cluster was entered, the HEb group was expanded to contain index samples and partitioned into 35 samples prior to patients entering the HEb cluster, 28 samples during patient presence in the HEb cluster and 15 samples after patients left the HEb cluster. For each patient, the Yue and Clayton dissimilarity index θ was calculated using taxa abundances in the last sample before entering the HEb cluster, the first sample in the cluster and the first sample after leaving the cluster (if such a sample existed). Samples showed more dissimilarity when leaving the cluster (θ = 0.11; Fig 4C) than when entering the cluster (θ = 0.27) compared to samples in the cluster. Interestingly, samples entering and leaving the clusters were the most similar (θ = 0.35), suggesting partial community restoration following transient presence in the cluster. Within the HEb group, the only significant taxa abundance changes upon entering the HEb cluster were a large drop in *Bacteroides* (Wilcoxon rank sum test, FDR < 0.003) and the expected large increase in *Enterobacteriaceae* (FDR = 0.01). The abundances of these taxa subsequently returned to near pre-HEb values upon leaving the cluster. Collectively, these analyses suggested a possible role for *Bacteroides* in opposing recurrent infection.

Transient presence in the HEb cluster was not associated with a concurrent or future increase in the abundance of *Peptostreptococcaceae* (Fig 4D), the family containing *C*. *difficile*. In fact, *Enterobacteriaceae* and *Peptostreptococcaceae* abundances were only weakly correlated within the entire HEb group (ρ = -0.01). Therefore, transient presence in the HEb cluster was hypothesized to temporarily create a metabolic environment that promoted CDI recurrence through an increase in *C*. *difficile* toxicity rather than *C*. *difficile* growth. To explore this hypothesis, the metabolite production capabilities of the HEb, HBo and HEc groups were compared. The metabolic signature of the partitioned HEb group (S9 Fig) was similar to that predicted when the HEb and HEc clusters were compared (S6 Fig, S6 Table) and included elevated synthesis of metabolites known to both induce (e.g. butyrate) and suppress (e.g. cysteine) the toxicity of *C*. *difficile* [76,77].

### FMT patient samples clustered by metabolic capability were associated with sample type

**Fig 5:**
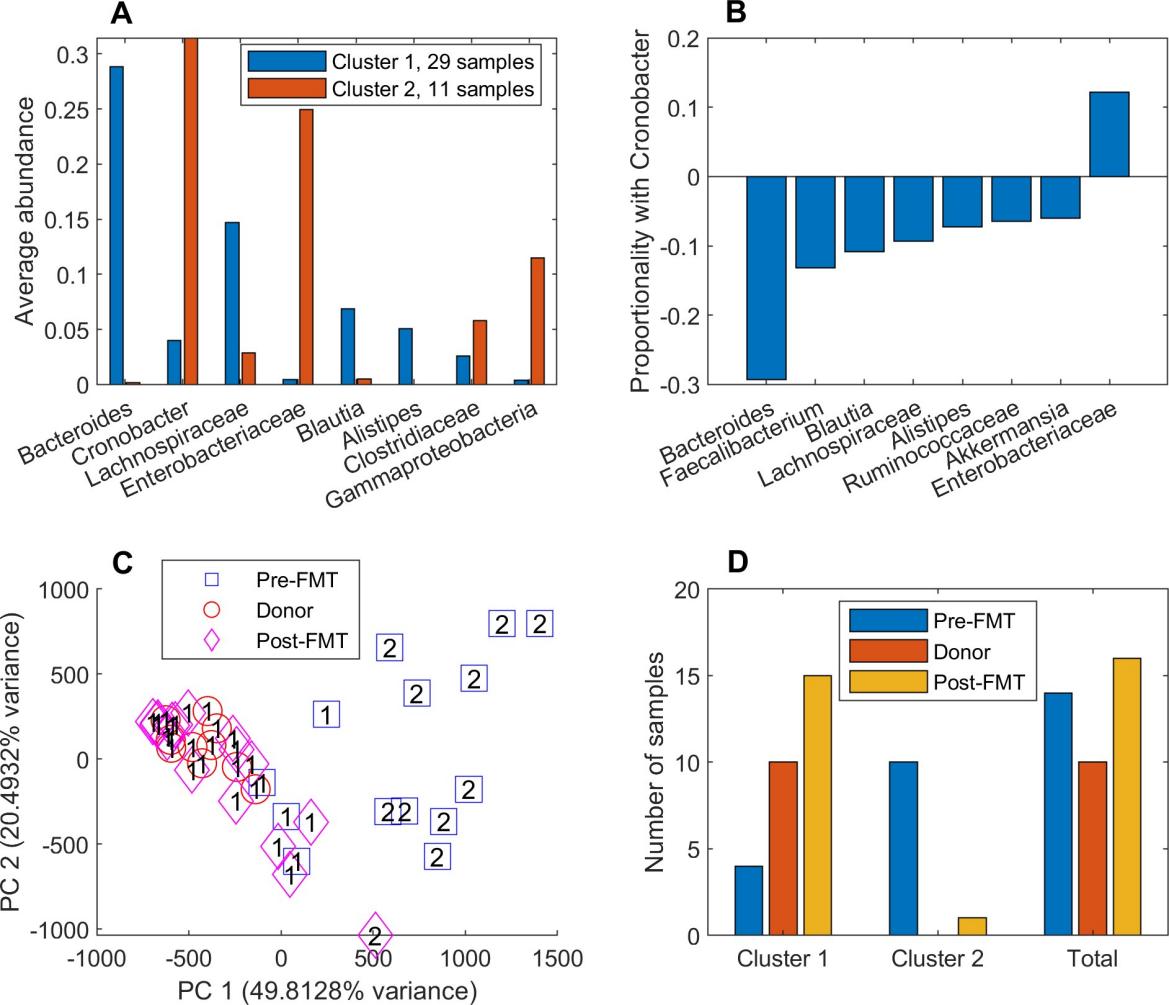
Clustering of 40 FMT samples using model-predicted metabolic capabilities. (A) Average taxa abundances across the samples in each cluster for taxa which averaged at least 5% in at least one cluster. (B) Correlations between *Cronobacter* and other taxa calculated from all 40 samples as measured by the proportionality coefficient ρ. The eight taxa with the largest |ρ| values are shown. (C) PCA plot of the model-predicted metabolic capabilities with each pre-FMT, donor and post-FMT sample labeled by its associated cluster number. (D) Number of pre-FMT, donor and post-FMT samples in each cluster and all 40 samples. Cluster 2 contained a disproportionately large number of pre-FMT samples (10/11) compared to the cluster 1 (4/29; p < 0.0001) and the entire FMT dataset (14/40; p = 0.0014).

Taxa abundance data derived from 40 stool samples representing 14 recurrent CDI patients undergoing FMT and from 10 of their donors [55] were modeled to investigate the community metabolic changes taking place upon FMT treatment. Model-predicted metabolic capabilities were partitioned with two clusters (silhouette value = 0.60) to generate one small cluster with elevated *Cronobacter*, *Enterobacteriaceae* and *Gammaproteobacteria* (averaged 67.9% across the 11 samples) and a second larger cluster with elevated *Bacteroides* and *Lachnospiraceae* (averaged 43.6% across the 29 samples; Fig 5A). Since *Cronobacter* belongs to the family *Enterobacteriaceae* and these two taxa averaged 56.4% across the 11 samples, the small cluster was considered to be dominated by *Enterobacteriaceae*. Consistent with these results, *Cronobacter* was most positively correlated with *Enterobacteriaceae* (proportionality coefficient ρ = +0.12) but negatively correlated with *Bacteroides* (ρ = -0.29) and several other taxa (*e*.*g*. *Lachnospiraceae*; Fig 5B) often reported to be CDI protective [73,74]. Similarly, *Bacteroides* was negatively correlated with *Enterobacteriaceae* (ρ = -0.21), *Gammaproteobacteria* and *Clostridiaceae* (S10B Fig), taxa which have been reported to be elevated in CDI [73,74,78].

When PCA was performed on the model-predicted metabolic capabilities, the *Enterobacteriaceae*-dominated cluster was clearly distinguishable and appeared to have an overrepresentation of pre-FMT patient samples (Fig 5C). In fact, this cluster contained a disproportionately large number of pre-FMT samples (10/11) compared to both the *Bacteroides*-elevated cluster (4/29; p < 0.0001) and the entire sample set (14/40; p = 0.0014; Fig 5D). Additionally, the *Enterobacteriaceae*-dominated cluster had a disproportionately small number of donor samples (0/11) and post-FMT patient samples (1/11) compared to the *Bacteroides*-elevated cluster (p = 0.038 and 0.027, respectively). The findings that the high *Enterobacteriaceae* (HEb) abundance cluster studied earlier contained a disproportionately large number of recurrent CDI samples (see Fig 2) and the high *Enterobacteriaceae* abundance cluster found here contained a disproportionately large number of pre-FMT samples provided additional support for the hypothesis that elevated *Enterobacteriaceae* is associated with recurrent CDI.

When similar analyses were applied directly to the abundance data, the dataset was partitioned into two clusters (silhouette value = 0.48) with one cluster having elevated *Cronobacter* and *Enterobacteriaceae* (averaged 35.9% across the 20 samples) and a second cluster having elevated *Bacteroides* and *Lachnospiraceae* (averaged 55.5% across the 20 samples; S10A–S10C Fig). Comparison of the two clusters generated with taxa abundances and with those generated with NMPCs generated a Rand value of 0.64, showing that two sets of FMT sample clusters were similar but also had samples clustered differently. The *Enterobacteriaceae*-elevated cluster generated with abundance data contained all the pre-FMT samples (14/20), representing large statistical differences with the *Bacteroides*-elevated cluster (0/20; p < 10^−5^) and the entire dataset (14/40; p = 0.0014; S10D Fig). By contrast, the *Bacteroides*-elevated cluster contained a disproportionally large number of donor samples (9/20) compared to the *Enterobacteriaceae*-elevated cluster (1/20). These results were consistent with those obtained from the model-predicted metabolic capabilities and collectively identified the pre-FMT samples as compositionally and functionally distinct from the donor and post-FMT samples.

### High Enterobactericeae abundance FMT cluster exhibited distinct bile acid and aromatic amino acid metabolism

**Fig 6:**
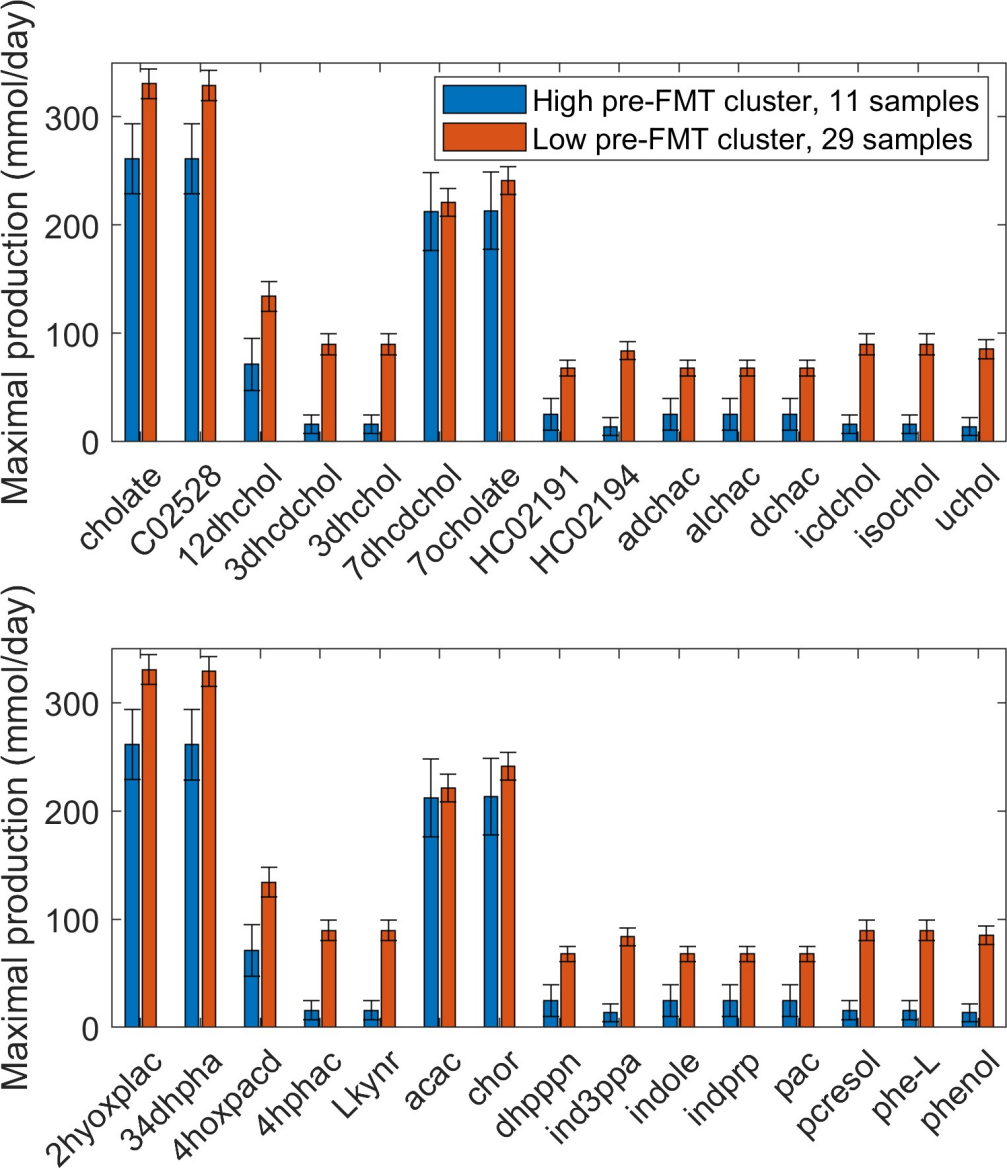
Net maximal production rates of bile acid and aromatic amino acid metabolites in the high *Enterobacteriaceae* and high *Bacteroides* abundance clusters generated from 40 model-predicted FMT samples. (A) Bile acid metabolites in which the average production rate was non-zero in at least one cluster. (B) Aromatic amino acid metabolites in which the average production rate was non-zero in at least one cluster. Error bars represent standard error of the mean. Metabolite abbreviations are taken from the VMH database (www.vmh.life). Full metabolite names, their associated metabolic pathways and numeric values for their average production rates in each cluster are given in S7 Table.

Predicted NMPCs of 411 exchanged metabolites were statistically analyzed to assess metabolic differences between the 40 samples clustered based on model-predicted metabolic capabilities. A comparison of the high *Enterobactericeae* abundance cluster (HEb-FMT, 11 samples) and the high *Bacteroides* abundance cluster (HBo-FMT, 29 samples) generated 58 differentially produced metabolites (S11 Fig). Only 22 of these 58 metabolites were identified as being differentially produced between the HEb and HBo clusters defined from model processing of CDI post-index samples (S5 Fig, S6 Table). Interestingly, 10 secondary BA metabolites and 4 AAA catabolic products were among the 36 newly identified metabolites. Therefore, BA and AAA metabolism in the HEb-FMT and HBo-FMT clusters were examined more carefully by comparing an array of secreted metabolites belonging to these pathways. The HEb-FMT cluster had decreased production of 15 BA metabolites (Fig 6A), including significantly reduced synthesis of 10 secondary BAs generally correlated with recurrent CDI [68,79,80]. By contrast, the HEb-FMT cluster had enhanced AAA metabolism as evidenced by elevated production of all 3 AAAs and 15 AAA catabolic products, including significantly increased synthesis of 8 degradation products (Fig 6B). Given that the HEb-FMT cluster was overrepresented in pre-FMT samples and underrepresented in donor and post-FMT samples, these predictions provided additional support for the hypothesis that community BA and AAA metabolism may be involved in CDI recurrence and FMT treatment.

When the same analysis procedure was applied to NMPCs clustered according to 16S-derived abundance data, 46 metabolites differentially produced between the *Enterobactericeae*-elevated and *Bacteroides*-elevated clusters were identified (S12 Fig). Overproduction of AAA catabolic products in the *Enterobactericeae*-elevated cluster continued to be pronounced, but differences in secondary BAs between the two clusters were no longer evident. The inability of the clustered abundance data to generate differential predictions of BA metabolism was attributed to the *Enterobactericeae*-elevated cluster containing 1 donor and 5 post-FMT samples in addition to all 14 pre-FMT samples. Therefore, clustering the samples according to model-predicted metabolic capabilities appeared to offer advantages for understanding community metabolic changes resulting from FMT.

## Discussion

An *in silico* pipeline was used to translate 16S-derived abundance data into sample-specific community models for investigating the metabolic determinants of recurrent CDI. The models generated sample-by-sample predictions of metabolite production rates that were used both to cluster samples according to their functional metabolic capabilities and to provide mechanistic insights into clusters exhibiting high recurrence. Community model predictions were dependent both on the taxonomic groups represented in the 16S data and the fidelity of individual taxa metabolic models. The CDI [13] and FMT [55] datasets used in this study captured taxonomic differences primary at the genus and family levels and therefore precluded modeling metabolism at the strain and species levels [53]. Despite this limitation, the pan-genome metabolic models used for community modeling allowed substantial differentiation of samples according to their functional capabilities.

Taxa abundance data and model-predicted metabolic capabilities were clustered to determine if the resulting clusters exhibited statistically significant differences between the number of recurrent CDI samples. No significant differences were observed when only index samples were tested, suggesting that community composition prior to CDI treatment may provide limited information about recurrence. By contrast, both abundance data and model-predicted metabolic capabilities derived from post-index samples identified high *Enterobacteriaceae*, low *Bacteroides* abundance clusters as having disproportionately large numbers of recurrent samples. Numerous studies have identified *Enterobacteriaceae* as positively associated and *Bacteroides* as negatively associated with primary CDI [72–74] and to a lesser extent with subsequent reinfection [81,82]. As in these experimental studies, the community models were able to establish a statistical relationship between high *Enterobacteriaceae*-containing samples and patient recurrence but could not definitively conclude if high *Enterobacteriaceae* was the cause or the result of recurrent CDI.

The analyses presented here suggest CDI recurrence is more dependent on community response to antibiotic therapy than on the community composition entering therapy. Indeed, first-line antibiotics for CDI treatment including metronidazole and vancomycin are known to collaterally target *Bacteroides* [83,84] while having little efficacy against *Enterobacteriaceae* [85–87]. Unfortunately, the metadata available for these samples only reported if the patient received antibiotic therapy prior to CDI diagnosis. Since next generation antibiotics such as fidaxomicin used for recurrent CDI are more specific for *C*. *difficile* and are known to spare *Bacteroides* [88,89], knowledge of which antibiotics were used to treat recurrent and nonrecurrent patients represented would enable additional analysis.

As compared to direct use of abundance data, an advantage of utilizing predicted metabolite production rates for sample clustering was that the high *Enterobacteriaceae* abundance (HEb) cluster contained more samples (28 vs. 15) representing more patients (22 vs. 11). The model-based cluster included samples with a high combination of *Enterobacteriaceae* and *Escherichia*, which have similar metabolic capabilities since *Escherichia* is a genus within *Enterobacteriaceae*. The capability to collapse samples with different compositions but similar metabolic features is useful when dealing with 16S-derived abundance data at several taxonomic levels, a common situation in human microbiome research.

Another benefit of quantifying metabolic capabilities through modeling was the ability to predict differentially synthesized metabolites across sample groups. When compared to a more taxonomically diverse cluster with elevated *Bacteroides* (HBo cluster) and no statistical difference in recurrence, the HEb cluster was predicted to have significantly reduced capabilities for secondary bile acid (BA) synthesis. These predictions were generally consistent with the established role of BA metabolism in recurrent CDI, as elevated primary BA and reduced secondary BA levels are known to be a disease signature [68,79,80]. The specific effects of individual BA metabolites are more nuanced, as the primary BA cholate induces induce germination of *C*. *difficile* spores, the primary BA chenodeoxycholate suppresses both germination and vegetative growth and the secondary BA deoxycholate induces germination but suppresses growth [73,90]. To achieve prediction at this level of granularity, the metabolic models would need to be constructed with 16S-derived abundance data at lower taxonomic levels or from whole genome metagenomic sequencing [91] since individual species and strains are known to have distinct BA metabolism [53].

Despite having no statistical difference in recurrence, a third cluster elevated in *Enterococcus* and to a lesser extent *Lactobacillus* (HEc cluster) had significantly reduced capabilities for secondary BA synthesis compared to both the HEb and HBo clusters. These predictions underscore the fact that recurrent CDI is a complex disease and not likely to be completely explained by a single factor such as community BA metabolism [55,74]. Interestingly, model-based analysis revealed aromatic amino acid (AAA) metabolism as a second putative mechanism underlying elevated recurrence in the HEb cluster. More specifically, this cluster was predicted to have significantly increased synthesis of numerous AAA degradation products compared to the two lower recurrence clusters. *Enterobacteriaceae* is thought to be largely responsible for AAA catabolism in the gut [41,92], and AAA synthesis has been implicated as a metabolic function protective against CDI [93]. *C*. *difficile* isolates have been shown to have highly variable AAA metabolisms [94], opening the possibility that *Enterobacteriaceae* interactions with *C*. *difficile* are isolate dependent. However, the 22 patients represented in the HEb cluster were reported to have been infected with at least nine different *C*. *difficile* ribotypes [13]. While evidence directly linking AAA metabolism and CDI is currently lacking, the modeling work presented here suggests that this putative connection could be a fruitful area for future experimental studies.

All samples from each patient with at least one sample in the HEb cluster were collected to allow longitudinal analysis of individual patients. This HEb group had a disproportionately large number of recurrent patients (19/22) compared to the larger patient population. HEb group patients exhibited compositionally variable communities that routinely switched between clusters, suggesting that transient presence in the HEb cluster could be sufficient for CDI recurrence. Since *Enterobacteriaceae* and *C*. *difficile* abundances had a very weak negative correlation within the HEb group, *Enterobacteriaceae* did not seem to support *C*. *difficile* vegetative growth but may have induced spore germination and/or enhanced toxicity of vegetative cells. As discussed above, the BA metabolite profile predicted for the HEb cluster was consistent with enhanced germination. *C*. *difficile* toxicity is thought to be regulated by a number of metabolites [76,77,95]. Two of the most potent regulators are toxicity-inducing butyrate and toxicity-suppressing cysteine, both of which were predicted to be elevated in the HEb cluster so as to have opposing effects. An intriguing but speculative possibility is that AAA degradation products from *Enterobacteriaceae* induced *C*. *difficile* toxicity.

To test consistency of model predictions derived from the CDI dataset, the *in silico* modeling pipeline was applied to 40 16S samples obtained from 14 FMT patients and 10 of their stool donors [55]. Clustering of model-predicted metabolic capabilities generated a cluster with a disproportionately large number of pre-FMT samples, suggesting distinct metabolic function compared to donor and post-FMT communities as has been reported [79,96,97]. This cluster had elevated *Cronobacter* and *Enterobacteriaceae* with very low *Bacteroides* abundances. Because *Cronobacter* is a member of *Enterobacteriaceae*, this cluster was identified as high *Enterobacteriaceae* abundance and was compositionally similar to the high recurrence HEb cluster found in the CDI dataset. A second cluster comprised mainly of donor and post-FMT samples was elevated in *Bacteroides* and *Lachnospiraceae* and compositionally similar to the HBo cluster identified from CDI samples. Consistent with these results, *Cronobacter* was found to be strongly positively correlated with *Enterobacteriaceae* and strongly negatively correlated with *Bacteroides* across the FMT dataset. These predictions agreed with findings that FMT tends to decrease the abundances of *Enterobacteriaceae* and other Proteobacteria [55,82,98] while increasing the abundances of *Bacteroides* and other health-promoting taxa such as *Lachnospiraceae*, *Blautia* and *Alistipes* [35,99,100].

The HEb cluster identified from FMT samples (HEb-FMT) was predicted to have reduced capabilities for synthesis of both primary and secondary BAs, while the HEb cluster derived from CDI samples (HEb-CDI) exhibited only reduced secondary BA synthesis. Unlike the pan-genome model of the family *Enterobacteriaceae*, the *Cronobacter* genus model lacked BA metabolic pathways because the necessary deconjugation and transformation genes have not been identified in *Cronobacter sakazakii*, the only member of this genus contained in the VMH database. The predicted difference in primary BA synthesis capabilities between *Enterobacteriaceae* in the CDI samples and *Cronobacter*/*Enterobacteriaceae* in the FMT samples demonstrate possible limitations of metabolic modeling at higher taxonomic levels and the potential value of more resolved sequence data. Despite these differences, the HEb-FMT cluster still exhibited reduced secondary BA levels observed in recurrent CDI [68,79,80] and resolved through FMT [79,80,101]. The HEb-FMT cluster also was predicted to have the capability for elevated AAA degradation including increased synthesis of the catabolic products phenylpyruvic acid, tyramine and tryptamine derived from phenylalanine, tryrosine and tryptophan, respectively. Because they also were elevated in the HEb-CDI cluster compared to high Bacteroides abundance (HBo-CDI) cluster, these three metabolites might make interesting experimental targets for their ability to induce germination and/or enhance toxicity of *C*. *difficile* clinical isolates.

Despite the ability of the proposed *in silico* workflow to identify high *Enterobacteriaceae*-containing communities as disproportionally recurrent and pre-FMT, model-based clustering did not result in idealized partitioning of patient samples. For example, the HEb-CDI cluster contained 3 nonrecurrent patients along with 19 recurrent patients, and the HEb-FMT cluster contained 1 post-FMT sample along with 10 pre-FMT samples. Similarly, the HEb clusters contained only subsets of all recurrent patients (22/66) and all pre-FMT samples (10/14). One possible explanation was that the likelihood of recurrence was dependent on the duration the transient community had an HEb cluster-like composition, as *Enterobacteriaceae* would require sufficient time to establish favorable metabolic conditions for *C*. *difficile* germination and/or pathogenicity. While intriguing, such speculation was impossible to test with the available dataset due to infrequent and irregular sampling. Perhaps the most plausible explanation for the clustering results is that recurrent CDI has a very complex disease etiology that depends on host-microbiota-environment interactions, both metabolic and non-metabolic. Therefore, the inability to perfectly classify patient recurrence based only on model-predicted metabolic capabilities was not surprising. However, the hypotheses that high *Enterobacteriaceae*-containing communities are more prone to recurrence and that recurrence may be partially attributable to the combination of disrupted BA and AAA metabolism seems worthy of further investigation through the type of integrated 16S sequencing and community modeling framework utilized in this study.

## Supporting information

S1 Table Metadata and normalized taxa abundances of 225 stool samples from 93 CDI patients based on data from [13]. (XLSX)

S2 Table Normalized taxa abundances of 40 stool samples from 14 FMT patients based on data from [14]. (XLSX)

S3 Table Total nutrient uptake rate bounds used in community metabolic models. (XLSX)

S4 Table Net maximal production rates of 411 exchanged metabolites calculated with 225 CDI community metabolic models. (XLSX)

S5 Table Net maximal production rates of 411 exchanged metabolites calculated with 40 FMT community metabolic models. (XLSX)

S6 Table Differentially produced metabolites between 3 clusters in 119 model-predicted post-index samples. (XLSX)

S7 Table Differentially produced metabolites between 2 clusters in 40 model-predicted FMT samples. (XLSX)

S1 Fig Schematic representation of the community metabolic modeling work flow. (A) Normalized taxa abundances were calculated from 16S rRNA data for CDI patient stool samples [13]. (B) Sample-specific community metabolic models were derived from the normalized taxa abundances using the Metagenomics Modeling Pipeline (mgPipe; [51]) within the MATLAB Constraint-Based Reconstruction and Analysis (COBRA) Toolbox. (C) mgPipe computed the net maximal production rate (NMPC) of every exchanged metabolite for each sample model. (D) The NMPC simulation data was subjected to machine learning and statistical tests to extract information on CDI recurrence.
(JPG)

S2 Fig Clustering of 90 index samples using 16S-derived abundance data. (A) Average taxa abundances across the samples in each cluster for taxa which averaged at least 5% of the total abundance. (B) Number of recurrent, nonrecurrent and total samples in each cluster and all 90 index samples. None of the clusters contained a disproportionate number of recurrent samples (Fisher’s exact test, p > 0.5). (C) PCA plot of the abundance data with each recurrent and nonrecurrent sample labeled by its associated cluster number.
(TIF)

S3 Fig Clustering of 119 post-index samples using 16S-derived abundance data. (A) Average taxa abundances across the samples in each cluster for taxa which averaged at least 5% of the total abundance. (B) Number of recurrent, nonrecurrent and total samples in each cluster and all 119 post-index samples. Cluster 2 contained a disproportionately large number of recurrent samples (14/15) compared to the cluster 1 (9/18; Fisher’s exact test, p = 0.009). (C) PCA plot of the abundance data with each recurrent and nonrecurrent sample labeled by its associated cluster number.
(TIF)

S4 Fig Clustering of 119 post-index samples with 2 to 6 total clusters using model-predicted metabolic capabilities. Average taxa abundances across the samples in each cluster for the 5 most abundant taxa across all samples: (A) 2 clusters; (B) 3 clusters; (C) 4 clusters; (D) 5 clusters; and (E) 6 clusters. (F) Fraction of recurrent samples in each cluster for 2 to 6 total clusters. The following clusters had a disproportionately large number of recurrent samples based on Fisher’s exact test: 2 total clusters, cluster 1 versus cluster 2 (p = 0.007); 3 total clusters, cluster 2 versus cluster 1 (p = 0.003); 4 total clusters, cluster 2 versus cluster 1 (p = 0.007); 5 total clusters, cluster 3 versus cluster 1 (p = 0.012); 6 total clusters, cluster 3 versus cluster 6 (p = 0.019), cluster 2 versus cluster 6 (p = 0.039).
(TIF)

S5 Fig Differentially produced metabolites in the high *Enterobacteriaceae* and high *Bacteroides* abundance clusters generated from 119 model-predicted post-index samples. Significant differences in metabolite production rates were determined by applying the Wilcoxon rank sum test (FDR < 0.05) to each metabolite across all samples in the two clusters. In addition to being statistically different, each metabolites shown had an average production rate > 50 mmol/day in at least one cluster and average production rates that differed between the clusters by at least 100%. Metabolite abbreviations are taken from the VMH database (www.vmh.life). Full metabolite names, their associated metabolic pathways and numeric values for their average production rates in each cluster are given in S6 Table.
(TIF)

S6 Fig Differentially produced metabolites in the high *Enterobacteriaceae* and high *Enterococcus* abundance clusters generated from 119 model-predicted post-index samples. Significant differences in metabolite production rates were determined by applying the Wilcoxon rank sum test (FDR < 0.05) to each metabolite across all samples in the two clusters. In addition to being statistically different, each metabolites shown had an average production rate > 50 mmol/day in at least one cluster and average production rates that differed between the clusters by at least 100%. Metabolite abbreviations are taken from the VMH database (www.vmh.life). Full metabolite names, their associated metabolic pathways and numeric values for their average production rates in each cluster are given in S6 Table.
(TIF)

S7 Fig Differentially produced metabolites in the high *Bacteroides* and high *Enterococcus* abundance clusters generated from 119 model-predicted post-index samples. Significant differences in metabolite production rates were determined by applying the Wilcoxon rank sum test (FDR < 0.05) to each metabolite across all samples in the two clusters. In addition to being statistically different, each metabolites shown had an average production rate > 50 mmol/day in at least one cluster and average production rates that differed between the clusters by at least 100%. Metabolites abbreviations are taken from the VMH database (www.vmh.life). Full metabolite names, their associated metabolic pathways and numeric values for their average production rates in each cluster are given in S6 Table.
(TIF)

S8 Fig Abundances of the top 7 taxa for all post-index samples of the 22 patients in the high *Enterobacteriaceae* abundance (HEb) group. All post-index samples from the 22 patients with at least one sample in the high *Enterobacteriaceae* abundance (HEb) cluster were grouped to generate the HEb group of 55 samples. The samples are denoted as XXX-Y where XXX is the patient ID and Y is the post-index sample number of that patient.
(TIF)

S9 Fig Differentially produced metabolites of the 22 patients in the expanded high *Enterobacteriaceae* abundance (HEb) group. The HEb group was expanded to contain pre-index and index samples to generate a dataset of 78 samples. These samples were partitioned into 35 samples prior to patients entering the HEb cluster, 28 samples during patient presence in the HEb cluster and 15 samples after patients left the HEb cluster. Significant differences in metabolite production rates were determined by applying the Wilcoxon rank sum test (FDR < 0.05) to each metabolite across all samples in the two groups compared (e.g. pre-HEb vs. HEb, pre-HEb vs. post-HEb, pre-HEb vs. post-HEb). In addition to being statistically different between at least two sample groups, each metabolites shown had an average production rate > 50 mmol/day in at least one group and average production rates that differed between the two groups by at least 100%. Metabolites abbreviations are taken from the VMH database (www.vmh.life). Full metabolite names and their associated metabolic pathways are given in S6 Table.
(TIF)

S10 Fig Clustering of 40 FMT samples using 16S-derived abundance data. (A) Average taxa abundances across the samples in each cluster for taxa which averaged at least 5% in at least one cluster. (B) Correlations between *Bacteroides* and other taxa calculated from all samples as measured by the proportionality coefficient ρ. The 9 taxa with the largest |ρ| values are shown. (C) PCA plot of the abundance data with each pre-FMT, donor and post-FMT sample labeled by its associated cluster number. (D) Number of pre-FMT, donor and post-FMT samples in each cluster and all 40 FMT samples. Cluster 2 contained a disproportionately large number of pre-FMT samples (14/20) compared to the cluster 1 (0/20; p < 0.00001) and the entire FMT dataset (14/40; p = 0.0014).
(TIF)

S11 Fig Differentially produced metabolites in the high *Enterobacteriaceae* (high pre-FMT) and high *Bacteroides* abundance (low pre-FMT) clusters generated from 40 model-predicted FMT samples. Significant differences in metabolite production rates were determined by applying the Wilcoxon rank sum test (FDR < 0.05) to each metabolite across all samples in the two clusters. In addition to being statistically different, each metabolites shown had an average production rate > 50 mmol/day in at least one cluster and average production rates that differed between the clusters by at least 100%. Metabolites abbreviations are taken from the VMH database (www.vmh.life). Full metabolite names, their associated metabolic pathways and numeric values for their average production rates in each cluster are given in S7 Table.
(TIF)

S12 Fig Differentially produced metabolites in the high *Enterobacteriaceae* (high pre-FMT) and high *Bacteroides* (low pre-FMT) abundance clusters generated from 40 FMT samples. Significant differences in metabolite production rates were determined by applying the Wilcoxon rank sum test (FDR < 0.05) to each metabolite across all samples in the two clusters. In addition to being statistically different, each metabolites shown had an average production rate > 50 mmol/day in at least one cluster and average production rates that differed between the clusters by at least 100%. Metabolites abbreviations are taken from the VMH database (www.vmh.life). Full metabolite names and their associated metabolic pathways are given in S6 and S7 Tables.
(TIF)
